# Supplementary material for: Unobtrusive measures of prejudice: Estimating percentages of public beliefs and behaviours
Source: PLoS One. 2021 Dec 22;16(12):e0260042. doi: 10.1371/journal.pone.0260042 (PMC8694410; doi:10.1371/journal.pone.0260042)
Supplement: Attachment — Submitted filename: PONELetter.docx [file pone.0260042.s002.docx]

**PONE-D-21-21013**

**Unobtrusive Measures of Prejudice: Estimating percentages of public beliefs and behaviours.**

Dear Dr. Haq

Thank you for submitting your email and review of our paper submitted to PLOS ONE. See below how we have reacted to each of the reviewers comments

1. Is the manuscript technically sound, and do the data support the conclusions?

Reviewer #1: Partly

Reviewer #2: Partly

2. Has the statistical analysis been performed appropriately and rigorously?

Reviewer #1: Yes

Reviewer #2: No

3. Have the authors made all data underlying the findings in their manuscript fully available?

Reviewer #1: Yes

Reviewer #2: No

4. Is the manuscript presented in an intelligible fashion and written in standard English?

Reviewer #1: Yes

Reviewer #2: Yes

5. Review Comments to the Author
)

**Reviewer #1:**

This article has an interesting topic. It has tried to show that people do not have accurate information about many economic and social facts of their society. Of course, in the introduction of the article, this issue is assumed. So the question is, what was the need for this research?
Of course, in my opinion, this research is necessary because we do not know which characteristics of society are wrong. That is, people's knowledge is far from reality.
This paper in terms of statistics is not complex and the method of work done is not so complicated. The positive point is that the research procedure is clearly described.

**Great News: many thanks**

I recommend the following tips to improve the quality of the article:
1. Explain the details of the survey in more detail. How many questions were there in the questionnaire? What tools have been used to measure intelligence? Were all the questions open-answer? When was the survey conducted? How do you know if the respondents were British citizens?

**We have answered all these questions in the revision and provided answers to each question.**

2- I think the authors can discuss at the end of the article a little about their understanding of the distance between public opinion and reality and express their theoretical ideas.

**We have been happy to do this and think it a good suggestion.**

3- That smarter people have more accurate information. It seems to be more controversial about this finding. PLease speculate more about this.

**We do not find it controversial but have happily speculated on why we found this result.**

**Reviewer #2:**

The goal of the study was to investigate prejudices about socio-demographic situation in Great Britain from a perspective of individual characteristics.
Here, I provide my comments:
1. I propose to rewrite an abstract: include some introduction, goal of the paper.

**OK...done as suggested.**

2. It is not clear based on what literature authors formulated their hypotheses.

**We have tried to explain this, but given that there is relatively little literature in this precise area we have noted where our hypotheses were speculative**

3. I recommend to say about nationality of your respondents in the section "Participants". Also, it seems vague to define citizenship based on nationality... How does it cause the results?

**See above...we have added these details and indeed speculated on how the population sample may impact the results**

4. I did not get how you found correlations in Table 3: could you please provide some example?

**Yes indeed:done as requested**

5. Page 8: "Three demographic factors (sex, age, education) and three ideological factors (religion, politics, life-after-death) were correlated with the 25 estimates. " - what is the Table for this sentence?

**We have clarified this point**

When you put the information about significance of the correlations: it is not clear what are the numbers in breaks (for example, "(sex: 6; age: 8; education: 0)")

**Yes: a good observation: we have now explained this**

6. When you found four new variables in PCA and then used them in regression you did not explain what exactly you used: accuracy of prejudices?

**We have now explained this point**

7. Page 11: "five factors were significant predictors of accuracy" - could you please explain this conclusion? It is not clear

**Ok....we have attempted to make this much clearer**

+ some additional remarks:

1. page 6: "There are indeed popular books and media programmes and blogs on peoples’ misunderstanding and misuse of statistics (Harford, 2020)." - why did you put this sentence there?

**Essentially to point out that people are very interested**

2. page 7: "The measure has been used in many studies (Bertsch & Pesta, 2009)." - but you mentioned only one.

**We have now referenced other papers.**

3. I recommend to put together Table 1 and Table 2.

**We have decided not to do this as we believe it is clearer to present the results as we have done initially**

Finally, I recommend publishing this manuscript after major revision (in case authors will be capable to answer all the questions and rearrange the manuscript).

**Thank you for your suggestions which were most helpful**
